# Gregariousness in the giant sloth *Lestodon* (Xenarthra): multi-proxy approach of a bonebed from the Last Maximum Glacial of Argentine Pampas

**DOI:** 10.1038/s41598-020-67863-0

**Published:** 2020-07-02

**Authors:** Rodrigo L. Tomassini, Claudia I. Montalvo, Mariana C. Garrone, Laura Domingo, Jorge Ferigolo, Laura E. Cruz, Dánae Sanz-Pérez, Yolanda Fernández-Jalvo, Ignacio A. Cerda

**Affiliations:** 1 INGEOSUR, Departamento de Geología, Universidad Nacional del Sur - CONICET, 8000 Bahía Blanca, Argentina; 2 Facultad de Ciencias Exactas y Naturales, Universidad Nacional de La Pampa, 6300 Santa Rosa, Argentina; 3 Departamento de Geodinámica, Estratigrafía y Paleontología, Facultad Ciencias Geológicas, Universidad Complutense de Madrid, 28040 Madrid, Spain; 4 Earth and Planetary Sciences Department, University of California Santa Cruz, Santa Cruz, CA 95064 USA; 5 Museu de Ciências Naturais, Secretaria do Meio Ambiente e Infraestrutura, Porto Alegre, 90690-000 Brazil; 6 Museo Argentino de Ciencias Naturales (MACN-CONICET), C1405DJR Buenos Aires, Argentina; 7 Departamento de Paleobiología, Museo Nacional de Ciencias Naturales (CSIC), 28006 Madrid, Spain; 8 Instituto de Investigación en Paleobiología y Geología (IIPG-CONICET), Universidad Nacional de Río Negro y Museo Carlos Ameghino, 8324 Cipolletti, Argentina

**Keywords:** Palaeoecology, Palaeoecology

## Abstract

Megamammals constituted an important component in the Pleistocene faunal communities of South America. Paleobiological and paleoecological studies involving different megamammal taxa have increased significantly in the last years, but there are still several poorly-known issues of its life history. In this work, we analyze an assemblage composed of 13 individuals of different ontogenetic stages, and possibly different sex, belonging to the giant ground sloth *Lestodon armatus* (Xenarthra, Folivora), recovered from Playa del Barco site (Pampean Region, Argentina). A dating of 19,849 years Cal BP allows assigning this assemblage to a period of the MIS (Marine Isotope Stage) 2 related to the end of the Last Glacial Maximum. Based on multiple lines of research (e.g. taphonomy, paleopathology, osteohistology, isotopy), we interpret the origin of the assemblage and diverse paleobiological and paleoecological aspects (e.g. social behavior, ontogenetic changes, sexual dimorphism, diseases, resource and habitat use, trophic relationships) of *L. armatus*. Evidence suggests that the assemblage was formed by a local single event of catastrophic mortality, which affected different members of a social group. This record represents the first accurate evidence of gregariousness for this ground sloth, providing new data on a poorly-known behavior among extinct Folivora.

## Introduction

In the last years there have been multiple studies on the Quaternary South American megamammals (estimated body mass ≥ 1,000 kg), including ground sloths^1–8^ (and references therein). Even so, there are several aspects of their life history that have not been addressed in detail, such as growth patterns, diseases, social behavior, habitat preference, feeding strategies, and trophic relationships with other mammals, both endemic to South America and immigrants from North America.

Sloths (Xenarthra, Folivora), one of the most conspicuous groups of mammals, include representatives of, at least, five monophyletic families, Bradypodidae, Megalonychidae, Megatheriidae^†^, Mylodontidae^†^, and Nothrotheriidae^†^^9,10^. Representatives of this clade were very abundant and diverse in the Quaternary terrestrial ecosystems in South America^6,11^. The extinction of ground sloths occurred in the late Pleistocene-early Holocene, along with that of the remaining megamammals^4,7^; it was proposed that the main causes of the extinction would be related to climate and environmental changes, diseases, human action, and combinations thereof^4,12–15^. They have no ecological analogues living today, as extant sloths are only represented by obligatory arboreal species of *Bradypus* (three-toed sloths) and *Choloepus* (two-toed sloths), which are restricted to the Neotropical rain forest^16^.

The ground sloth *Lestodon armatus* Gervais^17^ is the only valid species of the genus for the Quaternary^18^; it is the largest representative of Mylodontidae, with an estimated body mass of ~ 3,400–4,100 kg for adult individuals^19,20^. Remains assigned to this taxon are particularly abundant in late Pleistocene-early Holocene deposits of central Argentina, but there are also records in Brazil, Uruguay, Bolivia and Paraguay^18,21,22^.

The main goal of this work is to perform a multi-proxy analysis of a late Pleistocene assemblage constituted by several individuals of different ontogenetic stages assigned to the giant ground sloth *L. armatus*, from the Pampean Region of Argentina. Diverse taphonomic, pathological, osteohistological, and isotopic issues are herein evaluated in order to interpret and discuss paleoecological and paleobiological aspects of this species and the genesis of the assemblage.

A dating of 19,849 year Cal BP (using a *L. armatus* vertebra; see Ref.^13^) places the studied *L. armatus* assemblage at the end of the Last Glacial Maximum^23^. Although the timing of megamammal extinction in South America is not well-constrained, it seems that this phenomenon occurred since ~ 40 ka, with an accelerated pace starting at ~ 13.5 ka^4,13,24^. Therefore, this study provides novel information, based on multiple lines of evidence, on the life history of one of the largest members of the Quaternary fauna under an extinction scenario.

## Stratigraphical and sedimentological settings

**Figure 1:**
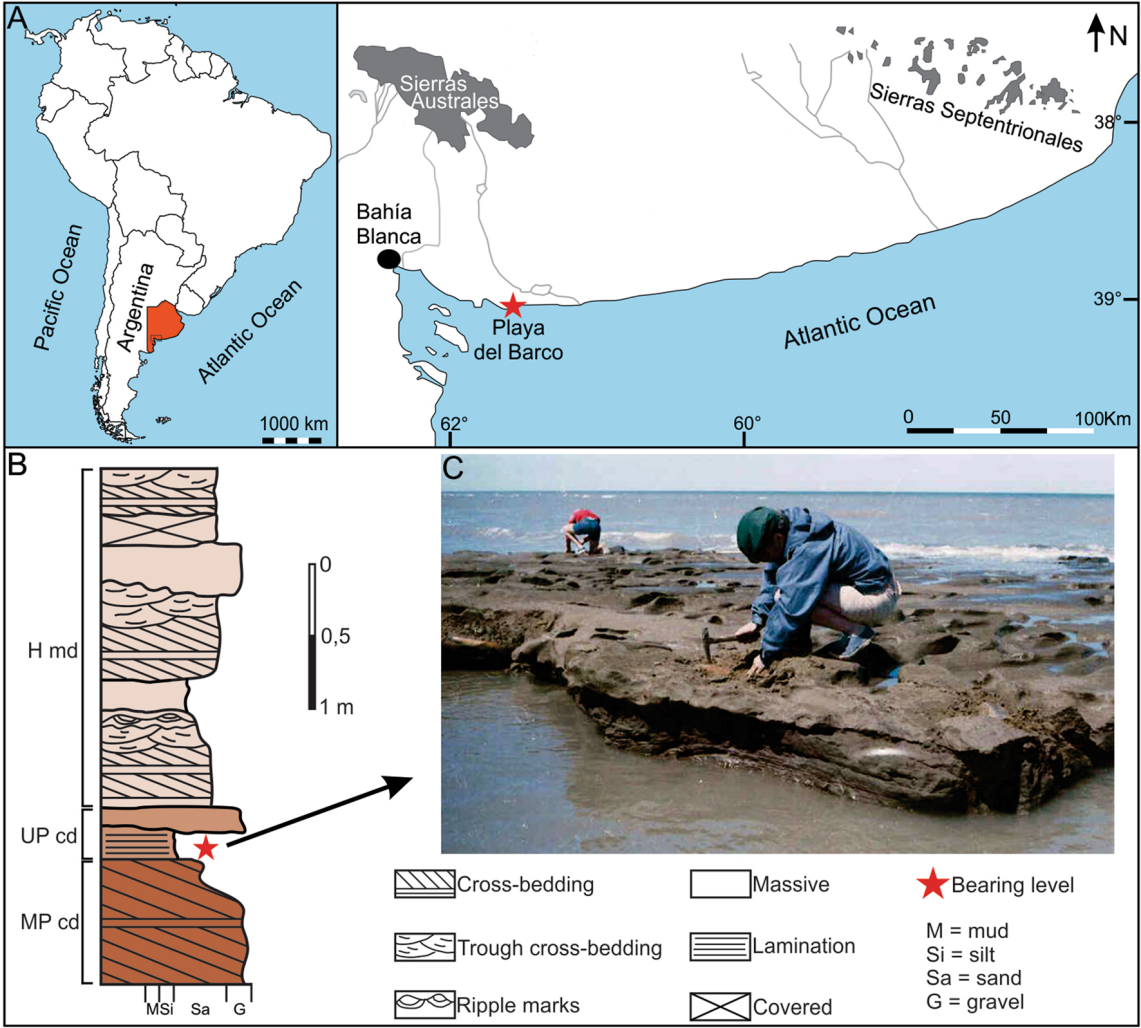
Geographical and stratigraphical settings of Playa del Barco site. (**A**) Map showing the location of the fossiliferous site in the coast of Buenos Aires Province (in red color). (**B**) Stratigraphic section of Playa del Barco site showing the different Quaternary levels. (**C**) View of the Upper Pleistocene bearing level; note the presence of several *Lestodon armatus* specimens. MP cd: Middle Pleistocene continental deposits; UP cd: Upper Pleistocene continental deposits; H md: Holocene marine deposits. Photo credits: Teresa Manera, Instituto Geológico del Sur INGEOSUR-CONICET, Argentina (CC BY open access license).

The Pampean Region of Argentina is characterized by several continental Pleistocene sites, some recognized from the nineteenth century by their paleontological richness. Mammal assemblages recovered in this area are of great importance and have constituted the basis to define the biochronostratigraphical schemes used in several regions of South America^4,25^ (and references therein). Playa del Barco (39°00′09″ S, 61°34′52″ W) is a fossiliferous locality in southwestern Buenos Aires Province, Argentina (Fig. 1A). There are scarce studies on its geology and paleontology due to the discontinuity of the outcrops, the reduced areal distribution, and the location in the current intertidal zone, which implies that they are usually covered by beach sand and are visible only during extreme low tides; however, several researchers highlighted the abundance and diversity of continental vertebrate remains^25^ (and references therein).

Sedimentary sequences generally are ~ 1 m (or less) thick and show both vertical and lateral facies variations (Fig. 1B). Both Pleistocene continental deposits and Holocene marine deposits have been recognized^26,27^ (Fig. 1B). All specimens of *L. armatus* studied herein were recovered from a tabular level, 15–25 cm thick, formed by dull yellow orange (10 YR 7/3) silty sand, massive or with diffuse parallel lamination, with subordinated 0.5–4 cm quartzitic clasts (Fig. 1B,C), which crops out in an area of ~ 500 m^2^. This level represents a high-density sheetflood deposit, accumulated in a floodplain environment; however, the difficulty to observe and analyze the outcrops prevents more detailed interpretations.

In addition to *L. armatus*, specimens of other 14 large and megamammals taxa were also recovered in this level (Supplementary Table S1). A radiocarbon dating made by Aramayo^29^, using a *L. armatus* vertebra from the fossil-bearing level, yielded an age of 16,440 ± 320 ^14^C years BP (Rafter Radiocarbon Laboratory, Institute of Geological & Nuclear Sciences Limited; New Zealand). Later, Prado et al.^13^ calibrated this value to calendar years before present and obtained a median age of 19,849 years Cal BP (20,242 years Cal BP and 19,455 years Cal BP). According to Ponce et al.^28^, the sea level during the Last Glacial Maximum was probably stable, approximately 120–140 m below the present level, generating a huge coastal plain along the Pampean Region; this situation conditioned the climatic and environmental characteristics of the area and, therefore, the faunal communities.

## Results

### Ontogenetic and anatomical representativeness

Most of the specimens (~ 65%) recovered from the Upper Pleistocene levels of Playa del Barco site correspond to *L. armatus*. The MNE (Minimum Number of Elements) of the studied *L. armatus* sample is 283 (Tables 1, 2). The MNI (Minimum Number of Individuals) is 13, including juvenile, adult, and senile individuals (Table 2). The MNI of the other recorded taxa is always less than 5 (Tomassini, personal observation). We do not discard a possible variation in the MNE and MNI of adult and senile (Table 2), as the differentiation between these two ontogenetic stages is based only in the presence/absence of pathologies, which are recorded in a limited number of postcranial elements (see below “Paleopathological analysis”).

**Table 1:** Minimum Number of Elements (MNE) and relative abundance (%Ri) values obtained for each skeletal element.

Skeletal elements	MNE	%MNE	%Ri
Skull fragments	4	1.41	–
Hemimandibles	14	4.95	53.8
Hemimaxillae	5	1.77	34.6
Isolated teeth	3	1.06	–
Scapulae	6	2.12	23
Humeri	4	1.41	15.3
Radii	3	1.06	11.5
Ulnae	4	1.41	15.3
Femora	5	1.77	19.2
Tibiae	8	2.83	30.7
Fibulae	4	1.41	15.3
Astragali	3	1.06	11.5
Metapodials	35	12.37	6.7
Phalanges	16	5.65	2.7
Atlas	9	3.18	69.2
Axis	1	0.35	7.6
Cervical vertebrae	12	4.24	18.4
Thoracic vertebrae	43	15.19	23.6
Lumbar vertebrae	5	1.77	12.8
Caudal vertebrae	41	14.49	12.6
Ribs	58	20.49	15.9
Total	283	100	20.6

**Table 2:** Taphonomic information of the *Lestodon armatus* sample from Playa del Barco site.

Quarry data	
Radiocarbonic age	19,849 years Cal BP
Size of accumulation	500 m^2^
Spatial density (specimens/m^2^)	~ 0.56
*Lestodon armatus* assemblage data	
MNE juvenile individuals	33
MNE adult individuals	209
MNE senile individuals	41
MNI juvenile individuals	4
MNI adult individuals	6
MNI senile individuals	3
Average relative abundance	20.6%
Bone modification data	
Articulation	Disarticulated but associated
Incomplete cranial elements	100%
Incomplete postcranial elements	79%
Weathering	18%
Abrasion	2%
Predation/scavenging	1%
Trampling	0%

A preliminary analysis shows some differences between the only two almost complete skulls (e.g. robustness, inclination of the occipital) and the mandibles (e.g. size, shape, and inclination of the caniniforms) of adult individuals, possibly reflecting the presence of two morphs. These differences were observed in other extinct species of ground sloths and suggested as indicators of sexual dimorphism^30–32^ (and references therein). Its record in the sample studied here could be an evidence of sexual dimorphism also in *L. armatus*. In this sense, Brambilla and Ibarra^33^ mentioned that the variability of the length from the first molariforms to the occipital condyles observed in skulls of *L. armatus* could indicate sexual differences; however, we could not evaluate this aspect because our two specimens do not preserve maxillae with molariforms.

**Figure 2:**
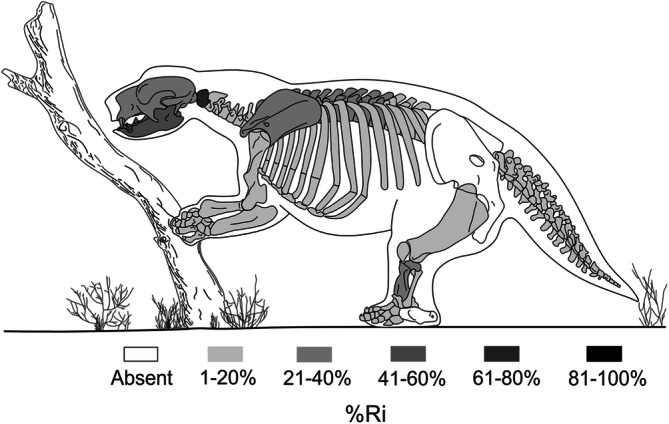
Relative abundance (%Ri) of *Lestodon armatus* skeletal elements from Playa del Barco site. Note that there are no skeletal elements with relative abundance values between 81 and 100%. No differentiation was made between left- and right-side elements.

Most skeletal elements are represented in the sample, but calcaneus, pelves, and sacral vertebrae are absent (Table 1). The average value of relative abundance is low, 20.6% (Tables 1, 2). There are no skeletal elements with relative abundance values ≥ 70%. The atlas displays the highest value, with 69.2%, followed by hemimandibles, hemimaxillae, tibiae, thoracic vertebrae, and scapulae. The remaining elements have values < 20% (Fig. 2, Table 1). All the groups proposed to evaluate the susceptibility of the skeletal elements to be transported by water flows^34–36^ are recorded in the sample.

### Taphonomic analysis

**Figure 3:**
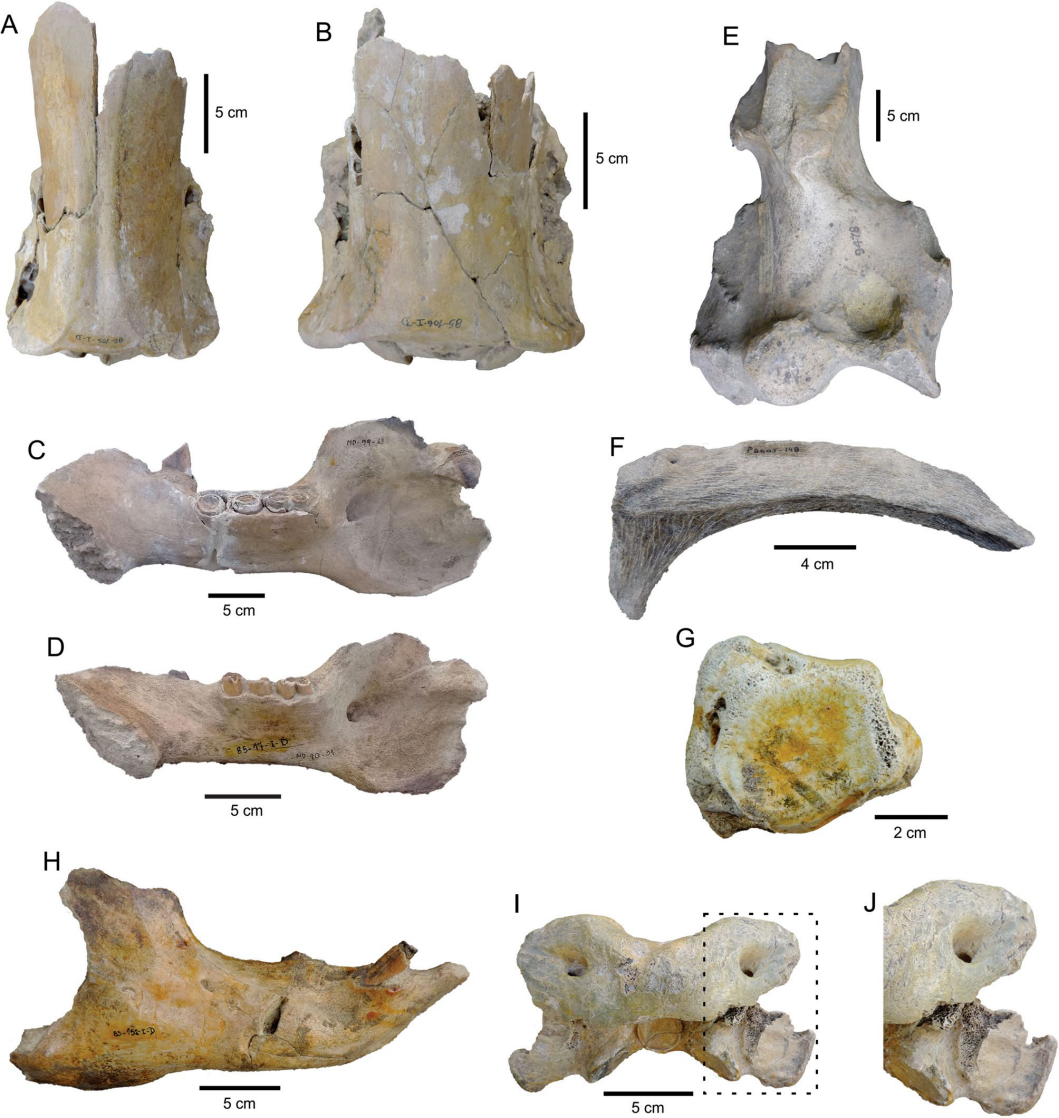
Taphonomic features of the *Lestodon armatus* specimens from Playa del Barco site. (**A**) MD-PDB-85-105, skull (dorsal view) with the anterior portion broken. (**B**) MD-PDB-85-106, skull (dorsal view) with the anterior portion broken. (**C**) MD-PDB-99-63, right hemimandible with complete dental series, lacking part of the posterior portion. (**D**) MD-PDB-85-17, right hemimandible with complete dental series, lacking part of the posterior portion. (**E**) MACN-PV-9478, distal portion of humerus showing a transversal fracture. (**F**) MD-PDB-05-148, fragment of rib with signs of weathering (slight splitting). (**G**) MD-PDB-85-150, metapodial with signs of abrasion (slight rounding). (**H**) MD-PDB-85-152, right hemimandible with crenulated edges in the posterior border, related with predators/scavengers activity. (**I**) MD-PDB-85-176, atlas with crenulated edges in the transverse processes, related with predators/scavengers activity. (**J**) Details of the marks on the vertebra shown in **I**.

Specimens of *L. armatus* were distributed in a small area (~ 500 m^2^), all of them disarticulated and isolated, but in close spatial proximity to one another (Table 2). Broken specimens predominate in the assemblage (Table 2). Complete specimens are mainly represented by metapodials and phalanges, but also include astragali, different long bones (humerus, ulna, tibia, and fibula), and thoracic and caudal vertebrae. Two almost complete skulls were recovered (Fig. 3A, B), although most of the cranial elements correspond to maxillae fragments. All the hemimandibles lack totally or partially the posterior portion (Fig. 3C, D). In most cases, both hemimaxillae and hemimandibles retain all teeth. Broken long bones mainly show smooth transverse fractures (~ 90%) (Fig. 3E), although stepped fractures (~ 10%) are also recorded. Ribs are mainly broken at the middle or distal portions, and show smooth transverse fractures, whereas most vertebrae do not preserve the processes. All scapulae are broken and represented by small portions.

Specimens with weathering (Table 2) show slight splitting parallel to the fiber structure, some of them reflect changes in the humidity, possibly related to water immersion and exposure events (Fig. 3F). Teeth (both isolated and retained in the alveoli) present slight splitting of dentine and orthodentine. Specimens with abrasion (Table 2) have slight rounding on the broken edges and ridges (Fig. 3G). Predation/scavenging marks (Table 2), observed in vertebrae and hemimandibles, are represented by crenulated edges (Fig. 3H–J). We do not recognize trampling marks.

### Paleophatological analysis

**Figure 4:**
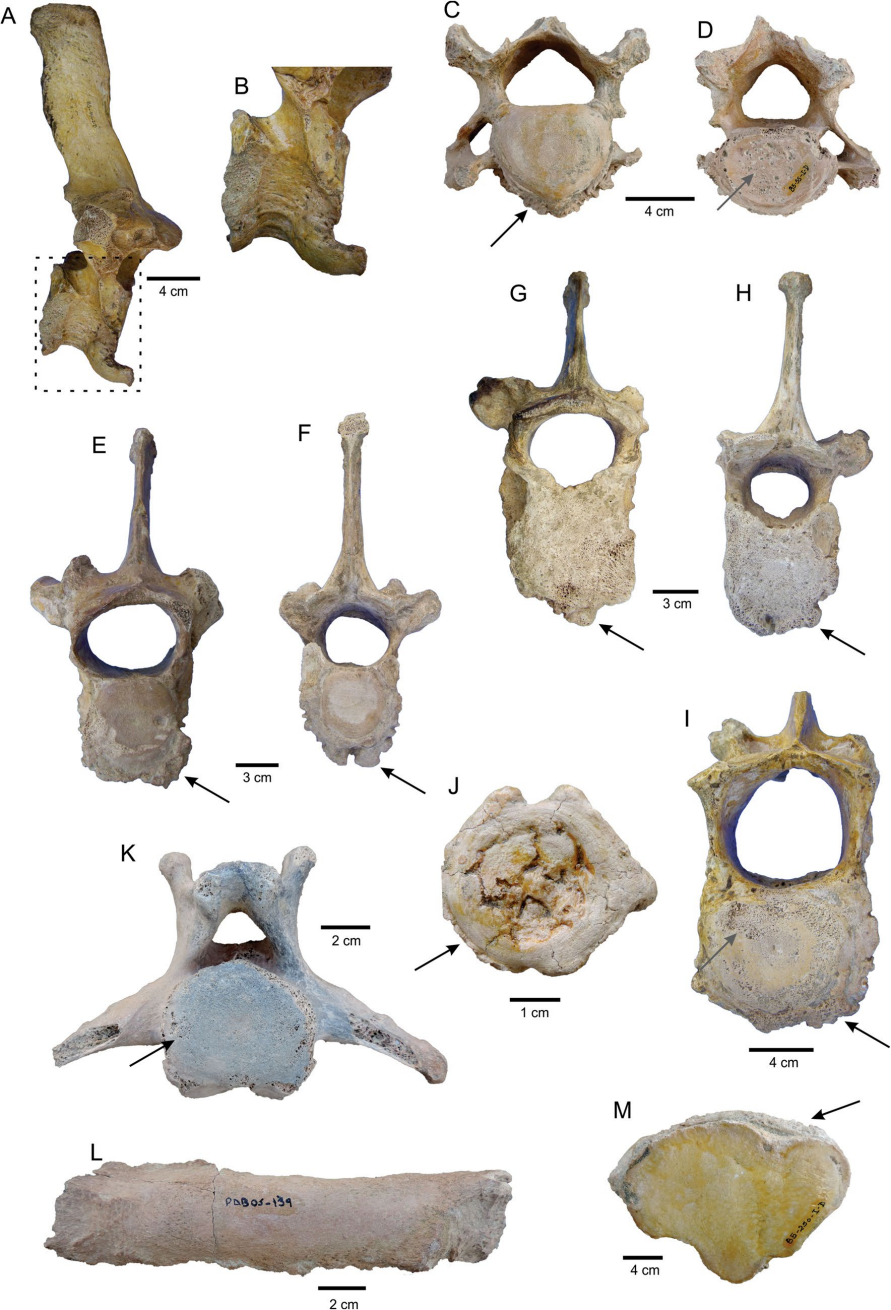
Paleopathological features of the *Lestodon armatus* specimens from Playa del Barco site. (**A**) MD-PDB-85-144, thoracic vertebra (right lateral view) with a very large osteophyte in the ventral margin of the vertebral body, possibly as part of an intervertebral bone bridge. (**B**) Detail of the osteophyte on the vertebra shown in A. (**C**,**D**) MD-PDB-85-55, cervical vertebra (**C**, anterior view; **D**, posterior view) with several osteophytes in the margins of the vertebral body (black arrow) and subchondral erosion in the posterior vertebral endplate (gray arrow). (**E**,**F**) MD-PDB-85-52, thoracic vertebra (**E**, anterior view; **F**, posterior view) with reduced height, several osteophytes in the margins of the vertebral body (black arrows), and discrete asymmetry in the neural arch. (**G**,**H**) MD-PDB-84-8, thoracic vertebra (G, anterior view; H, posterior view) with reduced height, several osteophytes in the margin of the vertebral body (black arrows), discrete arthrosis in the zygapophyses, and asymmetry in the neural arch. (**I**) MD-PDB-85-145, lumbar vertebrae (anterior view) with several osteophytes in the margin of the vertebral body (black arrow), subchondral erosion in the vertebral endplates (gray arrow), deformity of the vertebral body, and asymmetry of the neural arch. (**J**) MD-PDB-85-74, caudal vertebra (posterior view) with several osteophytes in the margin of the vertebral body (black arrow), and destruction of the posterior surface of the vertebral body. (**K**) MD-PDB-05-50, caudal vertebra (posterior view) with subchondral erosion (black arrow). (**L**) MD-PDB-05-139, rib with irregular bone surface and ossification of ligaments. (**M**) MD-PDB-85-250, metapodial with osteophytes (black arrow).

Pathological alterations are present in 41 skeletal elements (~ 14% of the sample). The affected elements include cervical, thoracic, lumbar, and caudal vertebrae, ribs, metapodials, and phalanges. Evidences of paleopathologies were also identified in long bones of *L. armatus* from other fossiliferous localities of the Pampean Region (Tomassini, personal observation). All types of vertebrae have osteophytes or bony spurs, which are mostly located in the margins of the vertebral body (Fig. 4A–H), but also in the costal articular facets of thoracic vertebrae; some osteophytes of thoracic and caudal vertebrae are very large and could have been part of intervertebral bone bridges (Fig. 4A,B). One cervical vertebra has subchondral erosion in the posterior vertebral endplate (Fig. 4D). Several thoracic vertebrae display reduced height, asymmetry in the shape/size of zygapophyses, transverse processes and neural arch, deformity of the spinous process (Fig. 4E–H), ossification of the ventral vertebral ligament, subchondral erosion in the vertebral endplates, and osteoporotic cancellous bone. Lumbar vertebrae show subchondral erosion in the vertebral endplates, deformity of the vertebral body (related to osteoporosis), and asymmetry of the neural arch (Fig. 4I). Caudal vertebrae also show destruction of the posterior surface of the vertebral body (Fig. 4J), and subchondral erosion in the vertebral endplates (Fig. 4K). Ribs display irregular bone surface and ossification of ligaments (Fig. 4L). Metapodials and phalanges present small osteophytes and irregular bone surface (Fig. 4M).

### Osteohistological analysis

**Figure 5:**
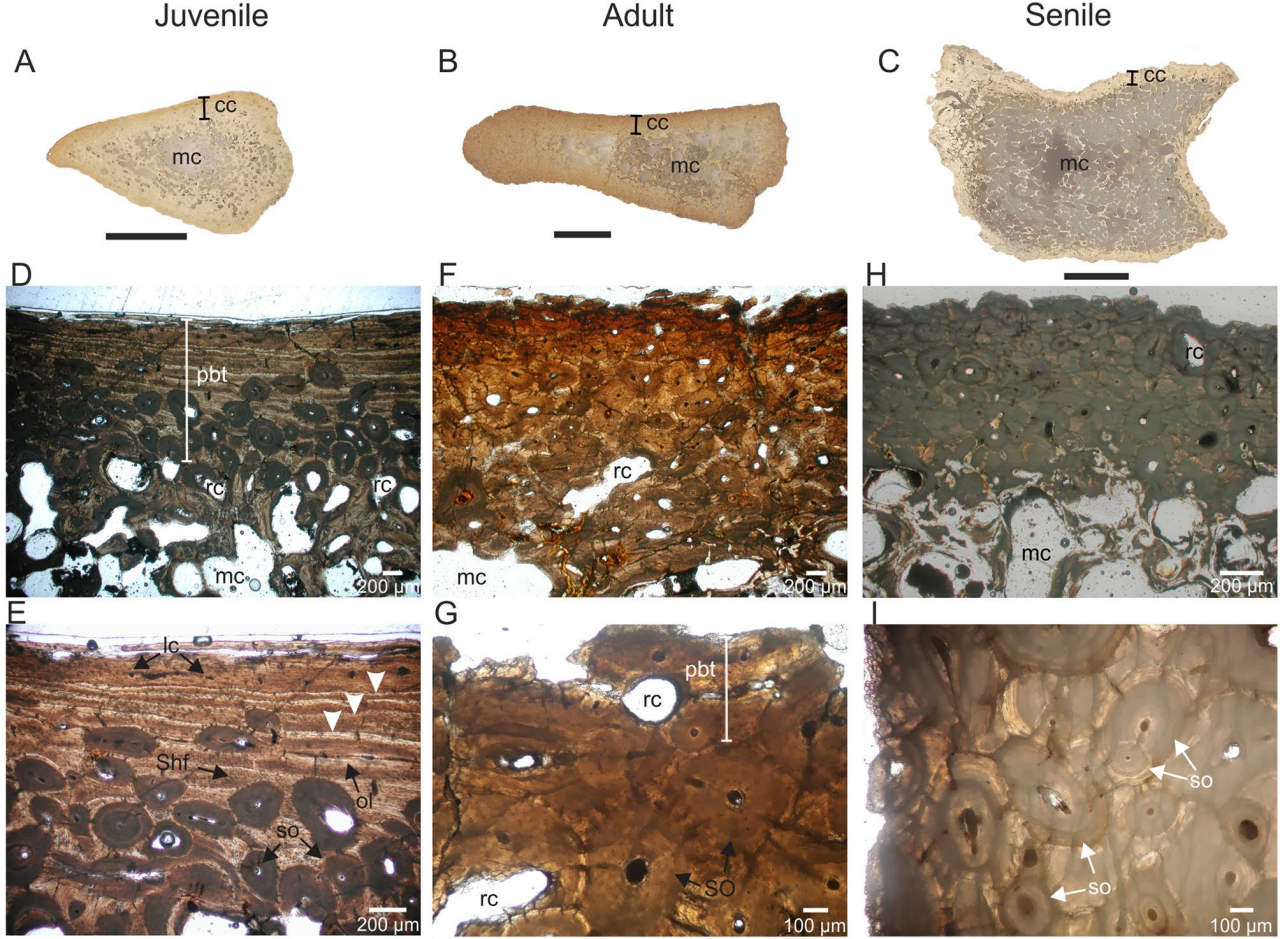
Osteohistological features of the *Lestodon armatus* specimens from Playa del Barco site. (**A**–**C**) General view of the spinous vertebral processes cross sections belonging to juvenile (MD-PDB-16-23), adult (MD-PDB- without catalogue number), and senile (MD-PDB-05-63) individuals. (**D**,**E**) details of the bone microstructure of the juvenile individual, in natural light, showing the primary bone tissue partially remodeled. Note the presence of three lines of arrested growth (LAGs, white arrows). (**F**,**G**) details of the bone microstructure of the adult individual, in natural light, showing the primary bone tissue (as a remnant) intensely remodeled. (**H**–**I**) details of the bone microstructure of the senile individual, in natural light, showing the primary bone tissue completely remodeled. cc: compact cortex. *lc* longitudinal channels, *mc* medullary cavity, *ol* osteocyte lacunae, *pbt* primary bone tissue, *rc* resorption cavity, *Shf* Sharpey’s fibers, *so* secondary osteon. Black scale bars = 1 cm.

Spinous vertebral processes of juvenile, adult, and senile individuals show a compact cortex surrounding a medullary cavity constituted by trabecular tissue (Fig. 5A–C). Primary bone tissue is present in the compact cortex of both juvenile and adult individuals. The primary bone matrix grades from parallel fibered to lamellar bone tissue. Osteocyte lacunae exhibit elongated shapes. Vascularization is reduced, characterized by the presence of randomly arranged longitudinal channels. Primary bone tissue is well developed in the middle and outer portions of the juvenile individual (Fig. 5D,E), while in the adult individual it is restricted to a thin subperiosteal layer (Fig. 5F). Primary bone tissue of the juvenile individual includes abundant Sharpey’s fibers bundles and three lines of arrested growth (LAGs; Fig. 5E). Secondary remodeling of compact bone is recorded in both individuals, represented in some sectors by Haversian bone with at least three partly overlapping generations of secondary osteons and resorption cavities (Fig. 5D–G). Haversian bone is clearly more extensive in the adult individual than in the juvenile. The cortical bone of the senile individual is completely remodeled, formed by Haversian bone with, at least, three partly overlapping generations of secondary osteons and resorption cavities (Fig. 5H,I). Cancellous bone in all the individuals is formed by secondarily deposited lamellar bone tissue.

### Stable isotope analysis

**Figure 6:**
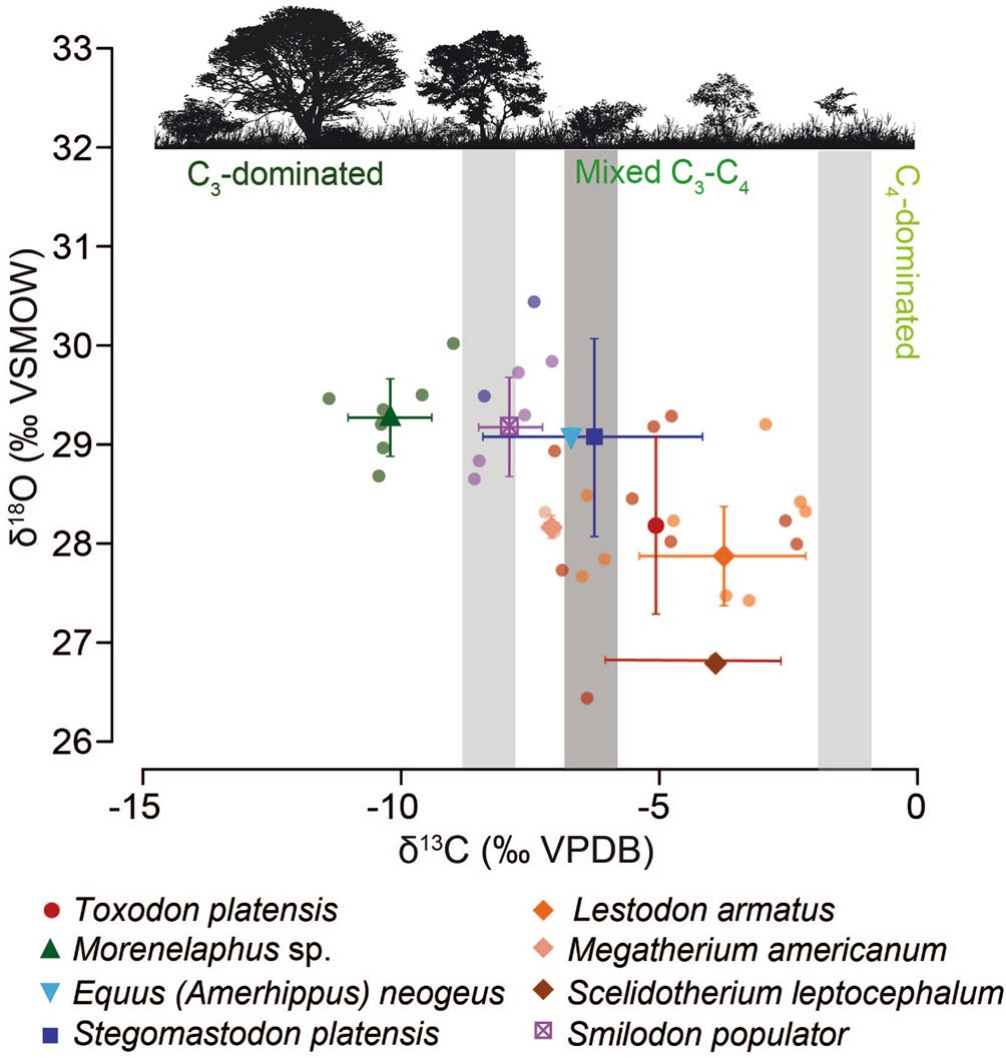
Raw and mean (± 1 standard deviation) δ^13^C (‰, VPDB) and δ^18^O (‰, VSMOW) values for different mammals from Playa del Barco site. The grey bars represent the vegetation δ^13^C cut-off values between a C_3_-dominated diet, a mixed C_3_-C_4_ diet, and a C_4_-dominated diet. The lightest grey denotes a δ^13^C bioapatite-diet enrichment of + 14.1‰ (according to Cerling and Harris^55^ values), whereas the darkest one corresponds to an enrichment of + 15.6‰ for xenarthrans (according to Tejada-Lara et al.^56^ values).

The mean δ^13^C value (± 1 standard deviation) of the herbivore mammals assemblage is − 6.3 ± 2.6‰ (VPDB; Vienna Pee Dee Belemnite) (Fig. 6, Supplementary Table S2). The highest mean δ^13^C value occurs in *L. armatus* (− 3.8 ± 1.6‰), whereas the lowest mean value occurs in *Morenelaphus* sp. (− 10.2 ± 0.8‰) (Fig. 6, Supplementary Table S2). Significant differences have been detected among taxa (F = 16.35, p < 0.001; Supplementary Table S3).

The mean bioapatite δ^18^O_CO3_ values (± 1 standard deviation) of the herbivore mammals assemblage is 28.6 ± 0.9‰ (VSMOW; Vienna Standard Mean Ocean Water), with the highest mean δ^18^O_CO3_ value recorded by *Morenelaphus* sp. (29.3 ± 0.4‰) and the lowest mean δ^18^O_CO3_ value depicted by *Scelidotherium leptocephalum* (26.8‰) (Fig. 6, Supplementary Table S2). The mean δ^18^O_CO3_ value of *L. armatus* is 27.9 ± 0.5‰. Significant differences also occur among taxa (F = 4.594, p = 0.003; Supplementary Table S3).

Values of δ^18^O_PO4_ are used here as a control for diagenetic alteration of biopatite, particularly in xenarthran orthodentine. The difference between δ^18^O_CO3_ and δ^18^O_PO4_ values (∆^18^O_CO3-PO4_ = δ^18^O_CO3_ − δ^18^O_PO4_) obtained for all taxa from Playa del Barco (Supplementary Table S4) are close to the obtained values from modern unaltered bioapatite^37,38^. Thus, stable isotope data from Playa del Barco sample can be used to assess past conditions.

## Origin and taphonomic history of the assemblage

Playa del Barco is a very rich fossiliferous locality of the Pampean Region, represented by several extinct large and megamammal taxa, both endemic to South America and immigrant from North America (Supplementary Table S1). An age of 19,849 years Cal BP^13^ allows assigning the studied assemblage from Playa del Barco to a period of the MIS 2 related to the end of the Last Glacial Maximum^23^, already within an extinction scenario of the South American megafauna^4,13,24^. This assemblage can be defined as a bonebed (sensu Rogers and Kidwell^39^). According to the classification of Eberth et al.^40^, it is a multitaxic, with high diversity, and monodominant bonebed, being *L. armatus* the most abundant taxon in terms of number of specimens and individuals. The sample of *L. armatus* includes several cranial and postcranial elements belonging to at least 13 juvenile, adult, and senile individuals, possibly both males and females. We performed here a detailed analysis of the *L. armatus* specimens from Playa del Barco site following several lines of evidence. This evaluation allowed us to interpret the origin and the possible taphonomic history of the assemblage.

The low average value of relative abundance reflects an important loss of skeletal elements; part of this loss could be linked with pre-burial processes. Based on the environmental context and the taphonomic evidence, we consider that most of the bones exposed in the surface would have been winnowed out towards other sectors by the high-density sheetflood generated during the flooding (i.e. events of overbank floods or rainfall) of the plain. All groups proposed to evaluate the susceptibility of skeletal elements to be transported by water flows^34–36^ were recorded; however, the values of relative abundance (Table 1) evidence a lower representativeness of the elements with high susceptibility (e.g. ribs, vertebrae, phalanges) with respect to the elements with low susceptibility (e.g. mandibles, maxillae), which suggest hydrodynamic sorting and differential loss of the elements.

The record of isolated specimens, but in close spatial proximity to one another, suggests that, during the exposure lapse in the surface, the carcasses became disarticulated and the bones sparsely mobilized and scattered, probably by water flows. The marked predominance of unweathered specimens (and weathered specimens showing very slight modifications), the scarcity of marks attributed to predation/scavenging, and the absence of clear trampling evidences would indicate that, in general, the exposure time was relatively short. Minimal abrasion in a few specimens reflects that the time of interaction between bones and sedimentary particles was short or that the intensity was very low, which is concordant with the environmental context of accumulation. The absence of anthropic activity signs is consistent with the proposed age (~ 13–12 ka) for the arrival of the first human groups to the Pampean Region of Argentina^7,41^. Taking into account the location of the fossiliferous site, in the current intertidal zone, we consider that the high degree of breakage obtained (with a clear predominance transversal fractures) could be related to the sea action during systematic current re-expositions of the outcrops occurred in extreme low tides.

The record of a high density of specimens distributed in a thin stratigraphic level restricted to a small area (~ 500 m^2^), belonging to several individuals of the same taxon, *L. armatus*, with different ontogenetic stages and possibly different sex, together with the observed taphonomic features (see Table 2), supports the interpretation of a local single event of catastrophic mortality to explain the origin of the *L. armatus* assemblage studied herein. This event would have been associated to a relatively short time of exposure in surface of elements that remained close to the place of death. It was not possible to determine the cause of death of the individuals, but an ontogenetic selective phenomenon can be discarded. According to Berger et al.^42^, assemblages of extant vertebrates originated by catastrophic death events are, in general, representative of living social groups. This type of monodominant assemblage is very useful to the study of paleobiological and paleoecological issues of a particular taxon^39,40^ (see below, “Gregarious behavior in *Lestodon armatus*”).

## Osteopathological interpretation

Different pathologies are identified in several postcranial elements. The asymmetry degree of neural arches, zygapophyses, and transverse processes reflects discrete osteoarthrosis. Marked height reduction, deformities, and high porosity of the vertebral body are modifications indicative of severe osteoporosis. The record of osteophytes and subchondral erosion in the vertebral bodies reflects the development of intervertebral discopathies. The presence of osteophytes in the costal articular facets of a thoracic vertebra suggests osteoarthrosis at the level of the costovertebral joints^43–45^. The identified intervertebral bone bridges would have prevented the sliding of a vertebra over another, avoiding injuries in the spinal medulla and nerves. On the other hand, this situation would also have significantly reduced the movements of the individual. Destruction of the posterior surface of the vertebral body in some caudal vertebrae is interpreted as osteochondritis dissecans. Both discopathies and osteochondritis dissecans of the caudal vertebrae could be related to a habit proposed for some ground sloths, which involves the use of the tail as a “third limb” to sit; this situation would produce an overload on that segment of the spine (see Ref.^44^).

The observed postcranial pathologies are frequent in large and megamammals, including extinct Folivora^43,44,46,47^. As it is mentioned previously^45^, particularly in the case of the vertebrae, these pathologies are compatible with individuals of advanced age and a huge body mass. This evidence improves the knowledge on the diverse diseases that affected the skeletal elements of the extinct ground sloths.

## Ontogenetic changes interpretation

The recorded microstructural features are consistent with the published descriptions on both extant and extinct Folivora, including *Lestodon*^48–52^. Some minor differences are fundamentally linked with the type of skeletal element analyzed, as most osteohistological studies are based on long bones and ribs.

The poorly vascularized parallel fibered to lamellar primary bone tissue, present in both juvenile and adult (as a remnant) individuals, indicates a slow apposition rate; this tissue is not recorded in the senile individuals because of the profuse Haversian remodeling. The record of dense Haversian bone in individuals of different ontogenetic stages suggests remodeling in progress of the primary bone tissue, until it is complete in the senile individual.

Straehl et al.^50^ mentioned that long bones of adult Xenarthra individuals are characterized by an important development of dense Haversian bone and highlighted that secondary remodeling is more important in large taxa than in small taxa, and particularly for folivorans, in extinct species more than in extant species. A proposal to explain this situation is that extinct Folivora had a more prolonged life span than extant representatives, which would favor the increase of size and loading, resulting in an extensive remodeling^50^. This idea is compatible with our results, which reflect a relative increase of the remodeling degree throughout the ontogeny; however, as we evaluated here a different skeletal element (thoracic vertebrae instead of long bones), other possible causes to explain the observed ontogenetic histological variations cannot be discarded.

According to several authors, LAGs would allow estimating the minimum age of an individual at the time of death^52–54^. Following this proposal, the identification of three LAGs in the juvenile individual suggests an age of, at least, three years; however, the age of this individual is possibly underestimated due to the loss of primary bone tissue by secondary remodeling and to the expansion of spongy tissue. The absence of LAGs in both adult and senile individuals would be linked with the extensive secondary remodeling (see Ref.^50^).

The obtained results reflect significant changes in the osteohistology of *L. armatus* during the ontogeny. In this sense, we observe a remarkable correspondence between the ontogenetic stages determined on the basis of macroscopic anatomical characters and the microstructural features.

## Niche occupation interpretation

The obtained values of δ^13^C from the Playa del Barco point to a preferential intermediate C_3_–C_4_ diet by most herbivorous taxa, being *Morenelaphus* sp. the only taxon consuming exclusively C_3_ plants (Fig. 6). Concerning *Lestodon armatus*, δ^13^C data (− 6.3 ± 1.6‰, VPDB) reflect a mixed C_3_–C_4_ diet or an intermediate diet between open C_3_ vegetation and mixed C_3_–C_4_ vegetation, which depends on the δ^13^C bioapatite-diet enrichment applied to xenarthrans (+ 14.1‰ according to Cerling and Harris^55^ or + 15.6‰ according to Tejada-Lara et al.^56^); in any case, there is a large variability among individuals, probably indicating some extent of dietary flexibility (Fig. 6). These results agree with collagen δ^13^C values obtained for *L. armatus* from Uruguay^57^, which point to foraging in relatively open areas, and support the proposal that considers *L. armatus* as a wide-muzzled sloth, mostly bulk-feeder, with a diet probably based on grass and herbaceous plants^58,59^.

When comparing different ground sloths from the Playa del Barco locality, *Megatherium americanum* depicts significantly lower δ^13^C values than *L. armatus* (t = 5.802, p < 0.001), pointing to a more browsing behavior for the former and the incorporation of food items from mixed C_3_-C_4_ areas for the latter (Fig. 6, Supplementary Table S2). In turn, the only analyzed sample of *Scelidotherium leptocephalum* shows a δ^13^C value (− 3.9‰, VPDB) similar to that depicted by *L. armatus*. According to Bargo et al.^58^, the marked hypsodonty of *S. leptocephalum* would support a digging behavior and the ingestion of abrasive food items. Differential dietary preferences and/or strategies among sympatric ground sloths may have eased the competition pressure and facilitated their coexistence^60^. On the other hand, the dietary flexibility depicted by *L. armatus* from Playa del Barco may have as well enabled the sympatry with endemic (e.g. *Toxodon platensis*) and immigrant (e.g. *Stegomastodon platensis*) herbivorous taxa, as no significant differences have been pinpointed among them (Fig. 6, Supplementary Table S2).

Unlike extant sloths, restricted to the Neotropical rain forest, extinct ground sloths were able to diversify in climates with arid and cool conditions, pointing to some mode of body temperature regulation^61^. Toledo et al.^61^ stated that ground sloths may have coped with climatic fluctuations by developing a hairy covering and by reaching large body sizes, which may have allowed them to better maintain a constant body temperature. This is supported by the difference between δ^18^O_CO3_ and δ^18^O_PO4_ values (∆^18^O_CO3-PO4_ = δ^18^O_CO3_ − δ^18^O_PO4_) obtained for ground sloths from Playa del Barco (Supplementary Table S4), similar to the difference observed in extant homeotherm mammals. This means that their bioapatite δ^18^O_CO3_ and δ^18^O_PO4_ values are likely related to their body water δ^18^O signal under a constant body temperature and therefore, it may have been directly routed from the δ^18^O value of ingested water. If so, the lack of significant differences with other herbivorous taxa from Playa del Barco would mean that *L. armatus* may have ingested water from the same hydrological sources (Supplementary Table S4).

## Gregarious behavior in *Lestodon armatus*

Gregariousness is a common behavior among living mammals, which favors the survival of the most vulnerable members against adverse intrinsic and extrinsic natural factors (e.g. predator attack, diseases, scarcity of resources such as food or water, adverse environmental and climatic conditions), parental care, and territory/resource defense, among others issues. For that reason, several species of herbivorous megamammals form large aggregations ^62,63^.

There are diverse biological and ecological traits of the extant megamammals that were also considered for extinct species, in order to shed more light on the relevance of this behavior in the past^64^. McDonald^29^ suggested, based on the remarkable differences in size and anatomy, that the extinct ground sloths probably had a more complex social structure than their extant relatives, which are solitary animals. However, specific studies including considerations on the social structure of extinct South American ground sloths are very scarce^65–68^.

**Figure 7:**
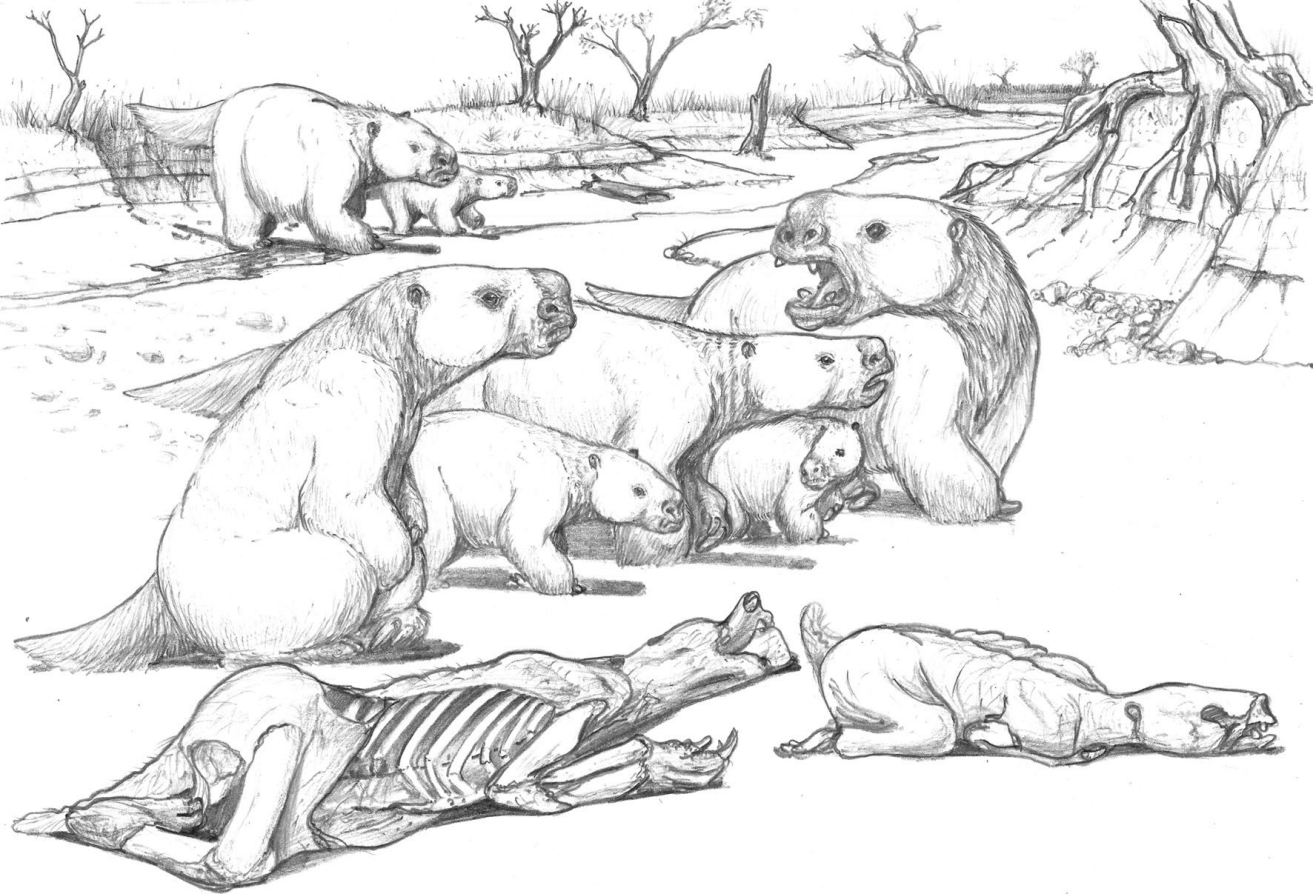
Artistic representation of a *Lestodon armatus* social group. Artwork by Pedro Cuaranta, Universidad Nacional del Nordeste, Argentina (CC BY open access license).

In this context, we highlight the significance of the *L. armatus* sample from Playa del Barco site. The identification of an assemblage formed by several individuals of different ontogenetic stages, possibly belonging to both males and females, likely originated by a local single event of catastrophic mortality, constitute and evidence of an intrinsic biogenic concentration (see Ref.^39^) that reflects a social behavior. Therefore, we interpret here that this mylodontid had, at least sometimes, gregarious habits forming intergenerational herds (Fig. 7). Other assemblages dominated by *L. armatus* have been reported for the Pleistocene of southern South America^2,69^, but in these studies there are not references on the possible social behavior of this species.

It is difficult to evaluate when and why gregarious behavior was acquired, because pre-Pleistocene record of *Lestodon*, and Mylodontidae in general, is very scarce. Although speculative, it could have been adopted or have been relevant during the Pleistocene in response to: (1) the occurrence of several glacial-interglacial cycles, which involved continuous and pronounced climatic and environmental changes^4,64^, and the consequent loss of habitats and/or temporal lack of resources; and (2) the impact linked with the most important pulses of the Great American Biotic Interchange. This event implied, on the one hand, the arrival of several herbivorous large and megamammals that would have generated competition for resources and habitats^70,71^. On the other hand, the interaction with new predators; isotopic analyses suggest that *L. armatus* was a probable prey of the carnivores *Smilodon populator* (present at Playa del Barco) and *Protocyon troglodytes*^71,72^.

## Conclusions

We report and analyze a bonebed, from the late Pleistocene of Pampean Region of Argentina, where the ground sloth *L. armatus* represents the dominant component. Sedimentological characteristics of the host level, density of specimens, number of individuals, ontogenetic representation, possible sexual dimorphism, and taphonomic features, allow us to interpret that the *L. armatus* accumulation was originated by a local single event of catastrophic mortality and represents, at least part of, a social group. This record is the first accurate evidence of gregariousness for this taxon, providing novel information on a poorly-known behavior among extinct Folivora.

This comprehensive multi-proxy study sheds new light on several paleobiological and paleoecological issues (e.g. social behavior, ontogenetic changes, sexual dimorphism, osteological diseases, resource and habitat use, trophic relationships) of a giant ground sloth endemic to southern South America. In an extinction scenario that began about ~ 40 ka ago, the age of the assemblage from Playa del Barco (19,849 years Cal BP) results interesting to evaluate the state of the megamammal communities, because it represents a moment: (1) linked with a key climatic event, the end of the Last Glacial Maximum; (2) posterior to the massive arrival of North American immigrants (herbivorous and carnivorous mammals); and (3) previous to the arrival of the first human groups. Finally, this type of analysis integrating different lines of evidence on bonebeds is critical to understanding the ecological relevance of the late Pleistocene megafauna and the possible impact of its extinction in the late Pleistocene-early Holocene.

## Material and methods

Analyzed specimens of *Lestodon armatus* are hosted in the paleontological collections of the Museo Municipal de Ciencias Naturales “Carlos Darwin” (Punta Alta, Argentina; acronym MD-PDB), and Museo Argentino de Ciencias Naturales “Bernardino Rivadavia” (Buenos Aires, Argentina; acronym MACN-PV). Taxonomic assignment was based on the identification of diagnostic characters and the comparison with other materials referred to this species. Most specimens come from old excavation, but they have precise information on their geographical and stratigraphical provenance. Some field data of the specimens (e.g. orientation, dip/trend) are lacking; however this situation did not prevent the interpretation on the taphonomic history of the assemblage.

Taking into account the poor knowledge on the ontogenetic growth of extinct Folivora, we assigned the specimens to three age classes (juvenile, adult, and senile) based on different macroscopic features. Relative size, dental wear degree, bone ossification (immature -trabecular- or mature bone), and fusion state (unfused or fused) of bone elements were used to differentiate juvenile and adult individuals. The presence of pathologies was the only criterion to differentiate adults and senile individuals. With respect to this, Ferigolo and Tomassini^45^ preliminarily interpreted that the pathologies present in the postcranial elements of *L. armatus* from Playa del Barco could be linked with old individuals.

Different indexes have been calculated in order to evaluate the anatomical representation in the sample. The MNE (Minimum Number of Elements) and MNI (Minimum Number of Individuals) were determined following Badgley^73^. In this case, the MNI was calculated considering the most frequent element independently for each identified age class (atlas for juveniles, left hemimandibles for adults, and 2nd thoracic vertebra for senile). Relative abundance of element i (%Ri) was calculated considering the representativeness of each element in the context of the MNI obtained, as follows: MNEi/(EixMNI) × 100, where MNEi is the minimum number of particular skeletal elements for the sample, and Ei is the expected number of these skeletal elements in a given individual, following Andrews^74^. No differentiation was made between left- and right-side elements. This index was used to interpret the loss of skeletal elements.

Skeletal elements were classified in different groups according to their susceptibility to be transported by water flows, following the proposals of Voorhies^34^, Behrensmeyer^35^, and Frison and Todd^36^. This evaluation is partially biased, because mentioned works evaluated extant taxa with very different body masses from *L. armatus*.

Features of the specimens were observed with the naked eye and through a binocular light microscope Leica MS 5 to interpret the taphonomic history of the assemblage. We evaluated: articulation (articulated, associated but disarticulated, and disarticulated and isolated; following Behrensmeyer^75^), breakage (complete or broken specimens), type of fractures in long bones (longitudinal, spiral, stepped, transverse; following Marshall^76^), weathering (unaltered, slight splitting and flaking, deep splitting and extensive flaking; modified from Behrensmeyer^77^), abrasion (unaltered, rounding, polishing; following Alcalá^78^), bioerosion marks (trampling, predator/scavenger; following Fernández-Jalvo and Andrews^79^). The spatial density was calculated considering the number of specimens by surface unit (following Behrensmeyer^75^).

Specimens were macroscopically analyzed in order to identify evidences of traumas, chronic diseases, or processes related to senility. Descriptions and diagnoses of the bone alterations follow Ferigolo^44^.

Thin transverse sections at the mid-length of spinous processes of three thoracic vertebrae were made in order to characterize the original bone microstructure and evaluate changes during the ontogeny. Thoracic vertebrae were the only element we had access to make thin sections, in which the different ontogenetic stages -juvenile, adult, and senile- could be identified; we assigned the ontogenetic stage to each specimen based on the macroscopic features mentioned above. Thin sections were made at the Laboratorio de Petrotomía of the INGEOSUR (CONICET), Departamento de Geología, Universidad Nacional del Sur, following standard petrographic techniques of Padian and Lamm^80^. They were observed and photographed using a Nikon Eclipse E400 POL petrographic microscope, under polarized light with a 1/4λ filter, with an incorporated digital camera. The osteohistological descriptions are mainly based on Francillon-Vieillot et al.^81^. We consider presence and distribution of primary and secondary tissues, vascularization pattern, form, density and disposition of osteocytes lacunae, presence and distribution of Sharpey’s fibers, and number and distribution of lines of arrested growth (LAGs).

We have evaluated the δ^13^C and δ^18^O data from different species of the Playa del Barco assemblage with the aim of unveiling the feeding behavior and habitat occupation of *L. armatus*. Tooth enamel was selected for notoungulates, perissodactyls, artiodactyls, proboscideans, and carnivorans, whereas orthodentine was used in the case of folivorans (Supplementary Tables S2 and S4). Sampling and technical protocols related to the stable isotope analysis of these samples are detailed in Domingo et al.^71^.

## Supplementary information

Supplementary file 1

## Data Availability

All data generated or analyzed during this study are included in the published article and in the Supplementary Information files.
