# Supplementary material for: Gregariousness in the giant sloth Lestodon (Xenarthra): multi-proxy approach of a bonebed from the Last Maximum Glacial of Argentine Pampas
Source: Sci Rep. 2020 Jul 2;10:10955. doi: 10.1038/s41598-020-67863-0 (PMC7331707; doi:10.1038/s41598-020-67863-0)
Supplement: Supplementary file 1 [file 41598_2020_67863_MOESM1_ESM.pdf]

**Supplementary Information for**

# Gregariousness in the giant sloth *Lestodon* (Xenarthra): multi-proxy approach of a bonebed from the Last Maximum Glacial of Argentine Pampas

Rodrigo L. Tomassini[1*], Claudia I. Montalvo[2], Mariana C. Garrone[1], Laura Domingo[3,4], Jorge Ferigolo[5], Laura E. Cruz[6], Dánae Sanz-Pérez[3], Ignacio A. Cerda[7], Yolanda Fernández-Jalvo[8]

[1]INGEOSUR, Departamento de Geología, Universidad Nacional del Sur - CONICET, Bahía Blanca 8000, Argentina. (rodrigo.tomassini@yahoo.com.ar*, garrone.mariana@gmail.com) *Corresponding author.

[2]Facultad de Ciencias Exactas y Naturales, Universidad Nacional de La Pampa, Santa Rosa 6300, Argentina. (cmontalvo@exactas.unlpam.edu.ar)

[3]Departamento de Geodinámica, Estratigrafía y Paleontología, Facultad Ciencias Geológicas, Universidad Complutense de Madrid, Madrid 28040, Spain. (ldomingo@ucm.es, dasanz01@ucm.es)

[4]Earth and Planetary Sciences Department, University of California Santa Cruz, Santa Cruz, California 95064, U.S.A.

[5]Museu de Ciências Naturais, Secretaria do Meio Ambiente e Infraestrutura, Porto Alegre 90690-000, Brazil. (jorgeferigolo@gmail.com)

[6]Museo Argentino de Ciencias Naturales (MACN-CONICET), Buenos Aires C1405DJR, Argentina. (cruzlaurae@gmail.com)

[7]Instituto de Investigación en Paleobiología y Geología (IIPG-CONICET), Universidad Nacional de Río Negro y Museo Carlos Ameghino, Cipolletti 8324, Argentina. (nachocerda6@yahoo.com.ar)

[8]Departamento de Paleobiología, Museo Nacional de Ciencias Naturales (CSIC), Madrid 28006, Spain. (yfj@mncn.csic.es)

**This PDF file includes:**

-Supplementary Table S1 (and references)

-Supplementary Table S2

-Supplementary Table S3

-Supplementary Table S4

**Table S1.** Mammals from Late Pleistocene levels of Playa del Barco site and their estimated body mass (EBM).

| Taxa | EBM (kg) |
|---|---|
| Order Folivora | |
| Family Megatheriidae | |
| *Megatherium americanum* | ~6,070[1,3,5] |
| Family Mylodontidae | |
| *Lestodon armatus* | ~3,400-4,100[1,2,5] |
| *Glossotherium robustum* | ~1,500-1,700[1,2,3,5] |
| *Scelidotherium leptocephalum* | ~850-1,050[1,2,3,5] |
| Order Cingulata | |
| Family Glyptodontidae | |
| *Glyptodon clavipes* | ~2,000[3,5] |
| *Glyptodon reticulatus* | ~410-860[1,3,4,5] |
| *Doedicurus clavicaudatus* | ~1,470-1,760[1,3,5] |
| *Panochthus* sp. | ~1,050-1,330[1,3,4,5] |
| Order Notoungulata | |
| Family Toxodontidae | |
| *Toxodon platensis* | ~1,640[1,3,5] |
| Order Litopterna | |
| Family Macraucheniidae | |
| *Macrauchenia patachonica* | ~1,000[1,3,5] |
| Order Proboscidea | |
| Family Gomphotheriidae | |
| *Stegomastodon platensis* | ~7,500-7,600[3,5] |
| Order Artiodactyla | |
| Family Cervidae | |
| *Morenelaphus* sp. | ~50[3,5] |
| Order Perissodactyla | |
| Family Equidae | |
| *Hippidion* sp. | ~500[1,3] |
| *Equus* (*Amerhippus*) *neogeus* | ~300[3,5] |
| Order Carnivora | |
| Family Felidae | |
| *Smilodon populator* | ~300-400[3,5] |

## References

1. Fariña, R. A., Vizcaíno, S. F., Bargo, M. S. Body mass estimations in Lujanian (Late Pleistocene-Early Holocene of South America) mammal megafauna. *Mastozoología Neotropical* **5**, 87–108 (1998).

2. Bargo, M. S., Vizcaíno, S. F., Archuby, F. M., Blanco, R. E. Limb bone proportions, strength and digging in some Lujanian (Late Pleistocene-Early Holocene) mylodontid ground sloths (Mammalia, Xenarthra). *J. Verteb. Paleontol*. **20**, 601–610 (2000).

3. Prevosti, F. J., Vizcaíno, S. F. Paleoecology of the large carnivore guild from the late Pleistocene of Argentina. *Acta Palaeontol. Polonica* **51**, 407–422 (2006).

4. Vizcaíno, S. F., Blanco, R. E., Bender, J. B., Milne, N. Proportions and function of the limbs of glyptodonts. *Lethaia* **44**, 93–101 (2011).

5. Fariña, R. A., Vizcaíno, S. F., De Iuliis, G. *Megafauna: giant beasts of Pleistocene South America*. Bloomington, Indiana University Press. 416 (2013).

**Table S2.** Summary of stable isotope dataset from Playa del Barco. Taxa, number of carbonate samples (# C), mean and standard deviation (SD) $\delta^{13}C$ (‰ VPDB), $\delta^{18}O_{CO3}$ (‰ VSMOW), number of phosphate samples (# P), mean and standard deviation (SD) $\delta^{18}O_{PO4}$ (‰ VSMOW), and difference between $\delta^{18}O_{CO3}$ and $\delta^{18}O_{PO4}$ values ($\Delta\ \delta^{18}O_{CO3}$-$\delta^{18}O_{PO4}$).

| **Taxa** | **# C** | **Mean $\delta^{13}C$ (‰ VPDB)** | **SD $\delta^{13}C$ (‰ VPDB)** | **Mean $\delta^{18}O_{CO3}$ (‰ VSMOW)** | **SD $\delta^{18}O_{CO3}$ (‰ VSMOW)** | **# P** | **Mean $\delta^{18}O_{PO4}$ (‰ VSMOW)** | **SD $\delta^{18}O_{PO4}$ (‰ VSMOW)** | **$\Delta\ \delta^{18}O_{CO3}$-$\delta^{18}O_{PO4}$** |
|---|---|---|---|---|---|---|---|---|---|
| All taxa | 36 | -6.3 | 2.6 | 28.6 | 0.9 | 23 | 19.7 | 1.2 | 8.9 |
| *Lestodon armatus* | 6 | -3.8 | 1.6 | 27.9 | 0.5 | 5 | 18.8 | 0.6 | 9.1 |
| *Megatherium americanum* | 2 | -7.1 | 0.1 | 28.2 | 0.1 | 2 | 18.7 | 0.1 | 9.5 |
| *Scelidotherium leptocephalum* | 1 | -3.9 | - | 26.8 | - | 1 | 18.0 | - | 8.8 |
| *Toxodon platensis* | 9 | -5.1 | 1.7 | 28.2 | 0.9 | 6 | 19.8 | 1.1 | 8.4 |
| *Stegomastodon platensis* | 5 | -6.3 | 2.1 | 29.1 | 1.0 | 4 | 20.7 | 1.2 | 8.4 |
| *Equus (Amerhippus) neogeus* | 1 | -6.7 | - | 29.1 | - | | | | |
| *Morenelaphus* sp. | 7 | -10.2 | 0.8 | 29.3 | 0.4 | 5 | 20.7 | 0.8 | 8.6 |
| *Smilodon populator* | 5 | -7.9 | 0.6 | 29.2 | 0.5 | | | | |

**Table S3.** ANOVA and Tukey post-hoc tests for $\delta^{13}C$ (‰ VPDB), $\delta^{18}O_{CO3}$ (‰ VSMOW) and $\delta^{18}O_{PO4}$ (‰ VSMOW) values among mammalian species from Playa del Barco.

| **$\delta^{13}C$ (‰ VPDB) ($F = 16.348$; $p < 0.001$)** | *Toxodon platensis* | *Megatherium americanum* | *Lestodon armatus* | *Stegomastodon platensis* | *Morenelaphus* sp. | *Smilodon populator* |
|---|---|---|---|---|---|---|
| *Toxodon platensis* | - | | | | | |
| *Megatherium americanum* | 0.455 | - | | | | |
| *Lestodon armatus* | 0.569 | 0.081 | - | | | |
| *Stegomastodon platensis* | 0.672 | 0.976 | 0.084 | - | | |
| *Morenelaphus sp.* | **< 0.001** | 0.115 | **< 0.001** | **0.001** | - | |
| *Smilodon populator* | **0.016** | 0.987 | **0.001** | 0.474 | 0.100 | - |

| **$\delta^{18}O_{CO3}$ (‰ VSMOW) ($F = 4.594$; $p = 0.003$)** | *Toxodon platensis* | *Megatherium americanum* | *Lestodon armatus* | *Stegomastodon platensis* | *Morenelaphus* sp. | *Smilodon populator* |
|---|---|---|---|---|---|---|
| *Toxodon platensis* | - | | | | | |
| *Megatherium americanum* | 1.000 | - | | | | |
| *Lestodon armatus* | 0.941 | 0.995 | - | | | |
| *Stegomastodon platensis* | 0.277 | 0.654 | 0.087 | - | | |
| *Morenelaphus sp.* | 0.051 | 0.375 | **0.013** | 0.994 | - | |
| *Smilodon populator* | 0.119 | 0.463 | **0.034** | 0.998 | 1.000 | - |

| **$\delta^{18}O_{PO4}$ (‰ VSMOW) ($F = 4.346$; $p = 0.013$)** | *Toxodon platensis* | *Megatherium americanum* | *Lestodon armatus* | *Stegomastodon platensis* | *Morenelaphus* sp. |
|---|---|---|---|---|---|
| *Toxodon platensis* | - | | | | |
| *Megatherium americanum* | 0.651 | - | | | |
| *Lestodon armatus* | 0.449 | 1.000 | - | | |
| *Stegomastodon platensis* | 0.560 | 0.153 | 0.052 | - | |
| *Morenelaphus sp.* | 0.424 | 0.111 | **0.028** | 1.000 | - |

**Table S4.** Stable isotope total dataset from Playa del Barco. Collection number, order, family, genus/species, analyzed tissue, $\delta^{13}C$ (‰ VPDB), $\delta^{18}O_{CO3}$ (‰ VPDB), $\delta^{18}O_{CO3}$ (‰ VSMOW), $\delta^{18}O_{PO4}$ (‰ VSMOW), and difference between $\delta^{18}O_{CO3}$ and $\delta^{18}O_{PO4}$ values ($\Delta$ $\delta^{18}O_{CO3}$-$\delta^{18}O^{PO4}$).

| **Collection number** | **Order** | **Family** | **Genus/Species** | **Analyzed tissue** | **$\delta^{13}C$ (‰ VPDB)** | **$\delta^{18}O_{CO3}$ (‰ VPDB)** | **$\delta^{18}O_{CO3}$ (‰ VSMOW)** | **$\delta^{18}O_{PO4}$ (‰ VSMOW)** | **$\Delta$ $\delta^{18}O_{CO3}$-$\delta^{18}O_{PO4}$** |
|---|---|---|---|---|---|---|---|---|---|
| MD-PDB-85-42 | Notoungulata | Toxodontidae | *Toxodon platensis* | Tooth enamel | -7.0 | -1.9 | 28.9 | | |
| MD-PDB-85-134 | Notoungulata | Toxodontidae | *Toxodon platensis* | Tooth enamel | -5.5 | -2.4 | 28.4 | | |
| MD-PDB-85-41 | Notoungulata | Toxodontidae | *Toxodon platensis* | Tooth enamel | -2.5 | -2.6 | 28.2 | 20.0 | 8.2 |
| Without signature | Notoungulata | Toxodontidae | *Toxodon platensis* | Tooth enamel | -4.8 | -2.8 | 28.0 | 19.8 | 8.2 |
| MD-PDB-85-279 | Notoungulata | Toxodontidae | *Toxodon platensis* | Tooth enamel | -4.8 | -1.6 | 29.3 | 21.2 | 8.0 |
| MD-PDB-90-26 | Notoungulata | Toxodontidae | *Toxodon platensis* | Tooth enamel | -6.9 | -3.1 | 27.7 | 20.0 | 7.7 |
| MD-PDB-97-13 | Notoungulata | Toxodontidae | *Toxodon platensis* | Tooth enamel | -2.4 | -2.8 | 28.0 | 19.6 | 8.4 |
| MD-PDB-7002 | Notoungulata | Toxodontidae | *Toxodon platensis* | Tooth enamel | -6.4 | -4.3 | 26.4 | 18.0 | 8.5 |
| MD-PDB-7001 | Notoungulata | Toxodontidae | *Toxodon platensis* | Tooth enamel | -5.1 | -1.7 | 29.1 | | |
| MD-PDB-97-1-1 | Folivora | Mylodontidae | *Scelidotherium leptocephalum* | Orthodentine | -3.9 | -4.0 | 26.8 | 18.0 | 8.8 |
| MD-PDB-90-30 | Folivora | Megatheriidae | *Megatherium americanum* | Orthodentine | -7.0 | -2.7 | 28.1 | 18.7 | 9.4 |
| MD-PDB-05-192 | Folivora | Megatheriidae | *Megatherium americanum* | Orthodentine | -7.2 | -2.5 | 28.3 | 18.8 | 9.5 |
| MD-PDB-99-63-2 | Folivora | Mylodontidae | *Lestodon armatus* | Orthodentine | -2.3 | -2.4 | 28.4 | 19.6 | 8.8 |
| MD-PDB-29-90-1 | Folivora | Mylodontidae | *Lestodon armatus* | Orthodentine | -4.7 | -2.6 | 28.2 | 18.9 | 9.3 |
| MD-PDB-29-90-2 | Folivora | Mylodontidae | *Lestodon armatus* | Orthodentine | -3.7 | -3.3 | 27.5 | | |
| MD-PDB-85-75 | Folivora | Mylodontidae | *Lestodon armatus* | Orthodentine | -6.5 | -3.2 | 27.6 | 18.5 | 9.2 |
| MD-PDB-05-113 | Folivora | Mylodontidae | *Lestodon armatus* | Orthodentine | -3.3 | -3.4 | 27.4 | 18.0 | 9.4 |
| MD-PDB-00-8 | Folivora | Mylodontidae | *Lestodon armatus* | Orthodentine | -2.2 | -2.5 | 28.3 | 19.0 | 9.3 |
| MD-PDB-85-274 | Proboscidea | Gomphoteriidae | *Stegomastodon platensis* | Tooth enamel | -7.4 | -0.5 | 30.4 | 21.9 | 8.5 |
| MD-PDB-85-261 | Proboscidea | Gomphoteriidae | *Stegomastodon platensis* | Tooth enamel | -8.4 | -1.4 | 29.5 | 20.5 | 9.0 |
| MD-PDB-85-19 | Proboscidea | Gomphoteriidae | *Stegomastodon platensis* | Tooth enamel | -6.1 | -3.0 | 27.8 | 19.1 | 8.8 |
| MD-PDB-08-20 | Proboscidea | Gomphoteriidae | *Stegomastodon platensis* | Tooth enamel | -3.0 | -1.7 | 29.2 | 21.2 | 8.0 |
| MD-PDB-99-43 | Proboscidea | Gomphoteriidae | *Stegomastodon platensis* | Tooth enamel | -6.4 | -2.4 | 28.5 | | |
| MD-PDB-90-17 | Perissodactyla | Equidae | *Equus (Amerhippus) neogeus* | Tooth enamel | -6.7 | -1.8 | 29.1 | | |
| MD-PDB-84-5 | Artiodactyla | Cervidae | *Morenelaphus* sp. | Tooth enamel | -10.4 | -1.7 | 29.2 | 21.1 | 8.1 |
| MD-PDB-84-5 | Artiodactyla | Cervidae | *Morenelaphus* sp. | Tooth enamel | -10.4 | -1.9 | 28.9 | 20.3 | 8.7 |

| | | | | | | | | | |
|---|---|---|---|---|---|---|---|---|---|
| MD-PDB-84-4 | Artiodactyla | Cervidae | *Morenelaphus* sp. | Tooth enamel | -11.4 | -1.4 | 29.4 | 19.9 | 9.6 |
| MD-PDB-84-4 | Artiodactyla | Cervidae | *Morenelaphus* sp. | Tooth enamel | -9.6 | -1.4 | 29.5 | 20.6 | 8.9 |
| MD-PDB-05-2 | Artiodactyla | Cervidae | *Morenelaphus* sp. | Tooth enamel | -9.0 | -0.9 | 30.0 | 22.0 | 8.0 |
| MD-PDB-05-110 | Artiodactyla | Cervidae | *Morenelaphus* sp. | Tooth enamel | -10.3 | -1.6 | 29.3 | | |
| MD-PDB-05-108 | Artiodactyla | Cervidae | *Morenelaphus* sp. | Tooth enamel | -10.4 | -2.2 | 28.7 | | |
| MD-PDB-83-99-1 | Carnivora | Felidae | *Smilodon populator* | Tooth enamel | -7.6 | -1.6 | 29.3 | | |
| MD-PDB-83-99-2 | Carnivora | Felidae | *Smilodon populator* | Tooth enamel | -7.1 | -1.1 | 29.8 | | |
| MD-PDB-83-99-3 | Carnivora | Felidae | *Smilodon populator* | Tooth enamel | -8.6 | -2.2 | 28.6 | | |
| MD-PDB-83-99-4 | Carnivora | Felidae | *Smilodon populator* | Tooth enamel | -8.5 | -2.0 | 28.8 | | |
| MD-PDB-83-99-5 | Carnivora | Felidae | *Smilodon populator* | Tooth enamel | -7.7 | -1.2 | 29.7 | | |
